# Resting State fMRI in Mice Reveals Anesthesia Specific Signatures of Brain Functional Networks and Their Interactions

**DOI:** 10.3389/fncir.2017.00005

**Published:** 2017-02-03

**Authors:** Qasim Bukhari, Aileen Schroeter, David M. Cole, Markus Rudin

**Affiliations:** ^1^Institute of Biomedical Engineering, University of Zurich and ETH ZurichZurich, Switzerland; ^2^Department of Psychiatry, Psychotherapy and Psychosomatics, University Hospital of PsychiatryZurich, Switzerland; ^3^Institute of Pharmacology and Toxicology, University of ZurichZurich, Switzerland

**Keywords:** fMRI, dual regression, brain network analysis, isoflurane, medetomidine, anesthesia, network interactions, rodent fMRI

## Abstract

fMRI studies in mice typically require the use of anesthetics. Yet, it is known that anesthesia alters responses to stimuli or functional networks at rest. In this work, we have used Dual Regression analysis Network Modeling to investigate the effects of two commonly used anesthetics, isoflurane and medetomidine, on rs-fMRI derived functional networks, and in particular to what extent anesthesia affected the interaction within and between these networks. Experimental data have been used from a previous study (Grandjean et al., 2014). We applied multivariate ICA analysis and Dual Regression to infer the differences in functional connectivity between isoflurane- and medetomidine-anesthetized mice. Further network analysis was performed to investigate within- and between-network connectivity differences between these anesthetic regimens. The results revealed five major networks in the mouse brain: lateral cortical, associative cortical, default mode, subcortical, and thalamic network. The anesthesia regime had a profound effect both on within- and between-network interactions. Under isoflurane anesthesia predominantly intra- and inter-cortical interactions have been observed, with only minor interactions involving subcortical structures and in particular attenuated cortico-thalamic connectivity. In contrast, medetomidine-anesthetized mice displayed subcortical functional connectivity including interactions between cortical and thalamic ICA components. Combining the two anesthetics at low dose resulted in network interaction that constituted the superposition of the interaction observed for each anesthetic alone. The study demonstrated that network modeling is a promising tool for analyzing the brain functional architecture in mice and comparing alterations therein caused by different physiological or pathological states. Understanding the differential effects of anesthetics on brain networks and their interaction is essential when interpreting fMRI data recorded under specific physiological and pathological conditions.

## Introduction

Analyzing adaptations of brain networks is becoming increasingly important for characterizing physiological or pathological states, or evaluating responses to therapeutic interventions. Parallel to the Human Connectome Project (Van Essen et al., 2013) there are considerable efforts to elucidate structural and functional connectivity also in rodents triggered by the expectation that rodent studies might provide valuable translational insight into mechanisms underlying FC and how these are altered during pathology. In functional magnetic resonance imaging (fMRI), functional connectivity across brain regions can be inferred from the temporal correlation of fluctuations in the baseline fMRI signal, i.e., under stimulus-free conditions. Several techniques have been suggested for analyzing resting state fMRI (rs-fMRI) data including seed-based analysis or dual regression. Seed-based analysis is straightforward, yet as univariate method considers each voxel independently, which implies that the approach considers only one effect at a time (Hampson et al., 2002). Seed-based analysis faces concerns related to the inherent biases of experimenter selection of seed regions (Cole et al., 2010). In addition, any network not associated to these seeds cannot be identified. The quality of seed-based analysis depends critically on the seed selection, which should be optimally adapted to the anatomical or functional brain areas. Therefore, regions are typically derived from a neuroanatomical atlas, which however may not be optimally adapted to the structural/functional unit for a specific subject due to anatomical variability and/or imperfect registration of the image data set to the atlas-based template. ICA and seed-based analysis are complementary approaches that have pros and cons. Seed-based analysis allows selection of fine-grained functional units; however, the use of fine-grained seeds is susceptible to errors related to registration. Also, it has been shown that biases inherent in the seed selection can result in a large variability in the results (Cole et al., 2010; Smith et al., 2014), which becomes crucial during network estimation. Nevertheless, if there is a strong prior hypothesis with regard to the involvement of specific brain regions, seed-based analyses using adapted seed masks are of great value. In contrast, regions derived from the ICA analysis are not subject to any anatomical constraints, which imply that given the limitations in sensitivity and intrinsic spatial resolution, they may not be ideally matched to structural/functional units as derived from a high-resolution brain atlas. Careful inspection of ICA results and removal of noise components becomes essential. Typically, ICA components are allocated to structural units in a *post-hoc* manner according to the best fit. ICA analysis does typically not produce fine-grained functional maps rendering them more robust against registration errors, at the expense of fine structure information. In the absence of a hypothesis ICA analysis appears appropriate, as it is purely data driven.

Dual Regression (DR) in combination with probabilistic independent component analysis (ICA) constitutes a multivariate approach for analyzing rs-fMRI data (Filippini et al., 2009) with spatial ICA maps being fed as input in to the DR pipeline. The DR approach (Filippini et al., 2009; Smith et al., 2014) first regresses the z-normalized group-IC spatial maps against the subject-specific 4D resampled datasets to give a set of subject-specific, variance normalized time courses for each component separately, and then—at a second-level of regression—these time-courses are regressed against the same 4D dataset to calculate a subject-specific set of spatial maps. The use of multivariate methods allows the simultaneous consideration of effects from all brain regions, i.e., the brain is treated as a fully connected network, or set of networks. ICA has been shown to produce reliable and comparable results both at the individual subject and the group level (Damoiseaux et al., 2006; Zuo et al., 2010). For in-depth network analysis, between-network interactions can also be considered by a comprehensive analysis of all ICA components (Smith et al., 2011). Graph theoretical approaches could be used to further analyze DR derived network information in order to structure them according to clusters (sub networks), nodes and edges. While frequently applied to human fMRI data, use of such approaches in small animal fMRI is still rather limited (Henckens et al., 2015; Grandjean et al., 2016). Network-based approaches have also been recently applied to mouse and rat rs-fMRI data, including prior parcellation into ICA components (Mechling et al., 2014; Liska et al., 2015).

Both medetomidine and isoflurane have been used in longitudinal fMRI experiments in rodents yielding robust BOLD response to external stimuli (Austin et al., 2005; Adamczak et al., 2010; Fukuda et al., 2013; Schroeter et al., 2014). As these agents involve different modes of actions, which affect both central and peripheral responses it is not surprising that fMRI responses were found to depend on the specific anesthetic used (Williams et al., 2010; Schroeter et al., 2014). Their differential effect is also reflected by anesthetic specific functional connectivity patterns (Williams et al., 2010; Grandjean et al., 2014). In particular, it has been reported that medetomidine, while yielding rather stable results in rats, decreases inter-hemispheric FC in mice (Jonckers et al., 2011, 2015; Nasrallah et al., 2014). In general, the optimal choice of anesthetic will depend on the specific problem to be addressed, i.e., should have minimal interference with the processes to be studied. The combination of complementary anesthetics may have synergistic effects and allow reducing the dose of the individual agents and thereby unwanted biochemical/physiological side effects (Fukuda et al., 2013; Grandjean et al., 2014).

In this study we evaluated the use of DR followed by graph theory based network analysis for detecting differences in mouse functional networks with respect to anesthesia-induced differences in physiological state. We analyzed the effects of two commonly used anesthetics and their combination, which have been shown to affect functional connectivity patterns in a drug-dependent manner (Grandjean et al., 2014). In particular, we focused on obtaining detailed interactions among networks and sub-networks of mouse brain functional architecture. We analyzed blood-oxygen level-dependent (BOLD) rs-fMRI data in terms of interacting fMRI networks by using partial correlation, which is thought to more closely represent brain functional principles than simple correlation of time courses extracted from individual seeds (Smith et al., 2011, 2014).

## Materials and methods

### Imaging

#### Animals, preparation, and anesthesia

The analysis is based on rs-fMRI data collected in an earlier study; we refer to Grandjean et al. (Grandjean et al., 2014), where experimental details have been described. In brief, female C57BL/6 mice of 10 to 15 weeks of age have been used for the study. For the rs-fMRI data collection mice had been intubated and artificially ventilated with an 80% air 20% oxygen mixture using a small animal ventilator (CWE, Ardmore, USA). Three groups of mice subject to different anesthesia protocols were studied: group 1 (*N* = 11) received 1% isoflurane administered via the ventilation mixture; group 2 (*N* = 13) an initial i.v bolus injection of 0.1 mg/kg medetomidine hydrochloride followed by a continuous infusion at a rate of 0.2 mg/kg/h of the drug; and group 3 (*N* = 8) received the combination of isoflurane and medetomidine with half the doses administered in groups 1 and 2, respectively. Rs-fMRI data have been acquired using a Bruker Biospec 94/30 small animal MR system (Bruker BioSpin MRI, Ettlingen, Germany) operating at 400 MHz (9.4 T) equipped with a four-element receive-only cryogenic phased array coil (Bruker BioSpin AG, Fällanden, Switzerland). Detailed acquisition parameters are given in Grandjean et al. (2014). For the current study, data have been downloaded from the central.xnat.org repository (Project ID: fMRI_ane_mouse; Grandjean et al., 2014). BOLD fMRI experimental data were acquired using a gradient-echo echo-planar imaging (GE-EPI) sequence: FOV = 23.7 × 14 mm^2^, MD = 90 × 60, yielding an in-plane voxel dimension of 263 × 233 μm, flip angle (FA) = 90°, bandwidth = 300 kHz, TR = 1000 ms, TE = 10 ms, NA = 1, yielding a temporal resolution of 1 s, with interleaved acquisition of slices. The duration of the image time series was 6 min. Mice were paralyzed in order to facilitate artificial ventilation and to eliminate motion artifacts during data acquisition. We analyzed reflexes and flinching behavior in non-paralyzed animals and did not detect any differences between animals anesthetized with either anesthetic nor did we detect any indication of pain.

### Data processing and statistical analysis

#### Preprocessing

All the preprocessing was performed using FSL's recommended preprocessing pipeline from FMRIB's Software Library (FSL version 5). Preprocessing included motion correction, removal of non-brain structures, high pass temporal filtering with sigma = 75.0 s, pre-whitening and global spatial smoothing using a filter with a 0.2 mm kernel. After the pre-processing the functional scans were aligned to the high-resolution template EPI scan using non-linear registration with 7 degrees of freedom as implemented in FLIRT, followed by nonlinear (FNIRT) warping (Jenkinson and Smith, 2001; Jenkinson et al., 2002)

#### ICA analysis and dual regression

We used FSL's MELODIC software for probabilistic independent component analysis (Beckmann and Smith, 2004). The multi-session temporal ICA concatenated (Concat-ICA) approach, as recommended for resting state data analysis (Beckmann and Smith, 2005; Beckmann et al., 2005), allowed the inputting of all subjects from all the groups in a temporally concatenated fashion for the ICA analysis. Concat-ICA yielded different components without the need for specifying any explicit time series model.

A total of 70 independent components (IC maps) were extracted from each analysis group. A mixture model approach was used to perform the inference on estimated maps. An alternative hypothesis test based on fitting a Gaussian/gamma mixture model to the distribution of voxel intensities within spatial maps (Beckmann and Smith, 2005) was used to threshold the IC maps. Out of the 70 independent components (IC maps) in each group, only 17 components on average were selected for each comparison, while the components that overlapped with vascular structures and ventricles were excluded from further analysis, however these components were still included as the regressors of no interest in the DR analysis. Similarly, components concentrated within the regions at the brain surface, which are prone to be affected by motion-related artifacts, were also excluded. Supplementary Figure 1 shows the removed ICA components.

We used DR (FSL 5.0.2.2) for between-subject analysis allowing for voxel-wise comparisons of rs-fMRI data (Filippini et al., 2009; Veer et al., 2010). We used unpaired *t*-tests to test for differences between anesthetic regimen conditions. Specifically, the design matrix was subject to [1 −1] contrasts to identify brain regions and networks displaying greater FC in one anesthetic condition relative to another.

Non-parametric permutation based inference analysis (Nichols and Holmes, 2002) was performed with subject-specific component spatial maps concatenated across subjects and submitted to voxel-wise between-subject analysis testing for effects of anesthetics on FC using FSL-randomize (Winkler et al., 2014). FSL's general linear model (GLM) was used to define contrasts based on unpaired *t*-test, testing for anesthesia effects among different groups. For each analysis we ran 5000 randomized permutations in line with the FSL default recommendations, while threshold-free cluster enhancement (Smith and Nichols, 2009) was used for statistical inference to validate the likelihood of extended areas of signal, which also takes into account information from neighboring voxels. TFCE enhances cluster-like structures but the image remains fundamentally voxelized. This cluster enhancement renders TFCE more sensitive than voxel-wise thresholding (Smith and Nichols, 2009). Correction for multiple comparisons across space was applied assuming an overall significance of *p* < 0.05 using permutation testing and TFCE. Bonferroni correction (*p* ≤ 0.05/17) was applied separately to each analysis depending on the number of components of interest (Tian et al., 2013).

#### Network modeling

FSLNets (FSL, 5.0.2.2) has been used for network modeling of rs-fMRI data. The data processing pipeline is depicted in Supplementary Figure 2. Different network matrix calculation methods have been applied. Full correlation (FC) estimates both direct and indirect connections, while partial correlation (PC) only estimates direct connections. We used L1 partial correlation method for Partial Correlation (PC) analysis, which yielded direct connections only (Smith et al., 2011). The PC matrices of the BOLD time courses of each component from dual regression were then clustered to form a dendrogram. These clusters were then used as input in to the GLM analysis and run through FSL-randomize (Winkler et al., 2014) to perform 5000 permutations to test for statistical significance. Edges, i.e., connections between network nodes showing statistically significant differences between the groups under consideration were obtained from GLM analysis. These significant network edges were then used to calculate network box plots (Supplementary Table 1 summarizes the values obtained through box plots) that take into account each edge and provide more information on differences in connectivity values between the groups. FSLNets was corrected for multiple comparisons with false discovery rate (FDR) using the same unpaired *t*-test design matrix as used previously for DR analysis.

## Results

### Dual regression confirmed results of seed-based analysis and identified additional components

**Figure 1:**
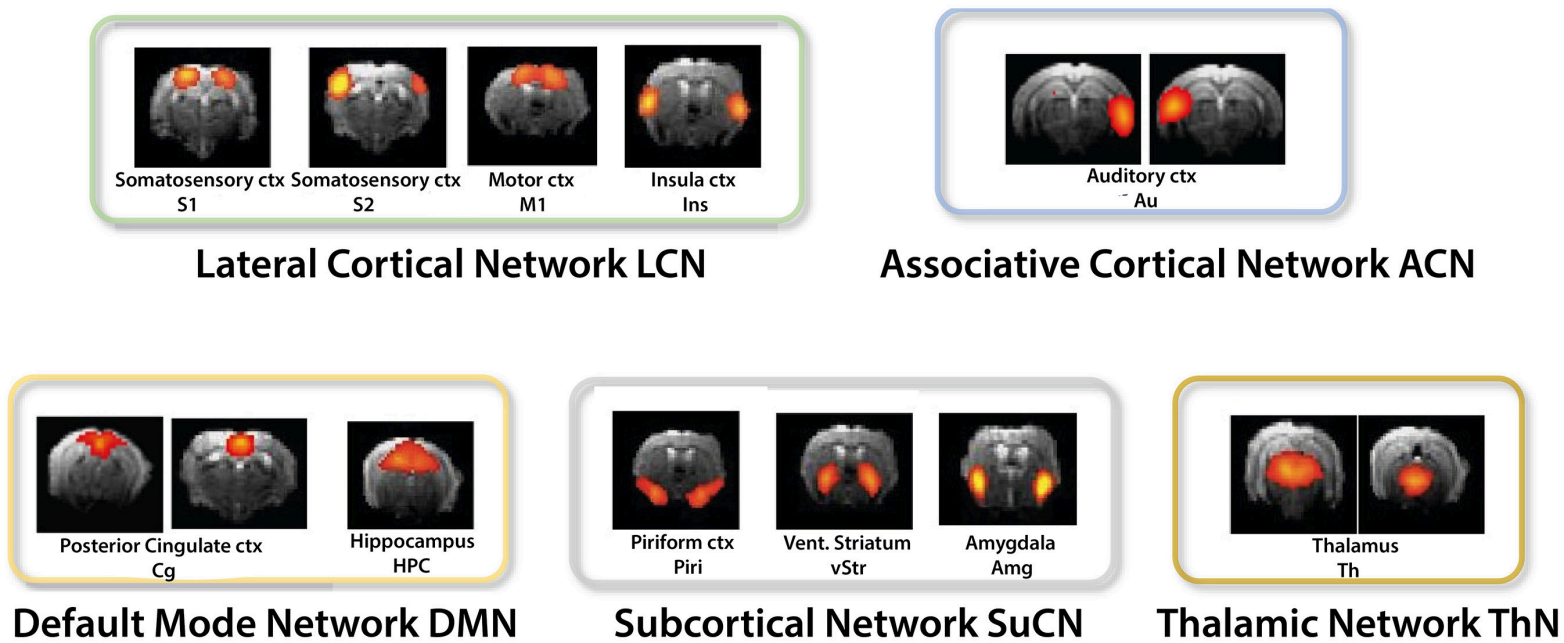
**ICA derived components were grouped into the lateral cortical (LCN), associative cortical network (ACN), default mode network (DMN), subcortical network (SuCN), and the thalamic network (ThN)**.

Of the 70 components derived from ICA, an average of 17 components (range 16–18) were retained for further analyses after discarding components at the brain surface and those involving vascular structures or ventricles. The number varied across individual analyses as a different number of components had to be discarded according to our selection criteria. Apart from auditory cortices, which appeared strictly lateralized, ICA derived components typically comprised bilateral homotopic brain areas (Figure 1, Table 1).

**Table 1:** **Regions identified in selected ICA components**.

		**Isoflurane**	**Medetomidine**	**med/iso**
Lateral Cortical Network (LCN)	Somatosensory cortex S1/S2	X	X	X
	Motor cortex M1	X	X	X
	Suppl motor cortex M2	X	X	X
	Insular cortex Ins	X	X	X
Associative Cortical Network (ACN)	Limbic cortex Lim	X	X	X
	Visual cortex Vis		X	
	Auditory cortex Au	X	X	X
Default Mode Network (DMN)	Prefrontal cortex PFC		X	
	Cingulate cortex Cg	X	X	X
	Dorsal hippocampus dHPC	X		
	Ventral hippocampus vHPC	X	X	X
Subcortical Network (SuCN)	Piriform cortex Piri	X	X	X
	Dorsal striatum dStr	X		
	Lateral striatum lStr			X
	Ventral striatum vStr	X	X	X
	Amygdala Amg	X	X	X
Thalamic Network (THN)	Dorsal thalamus dTh	X	X	X
	Ventral thalamus vTh	X	X	X
Extended SuCN	Hypothalamus HTH	X	X	X
	Globus pallidus GP	X	X	
	Olfactory tubercle OT	X		X

*Allocation of ICA components identified to major brain networks*.

**Figure 2:**
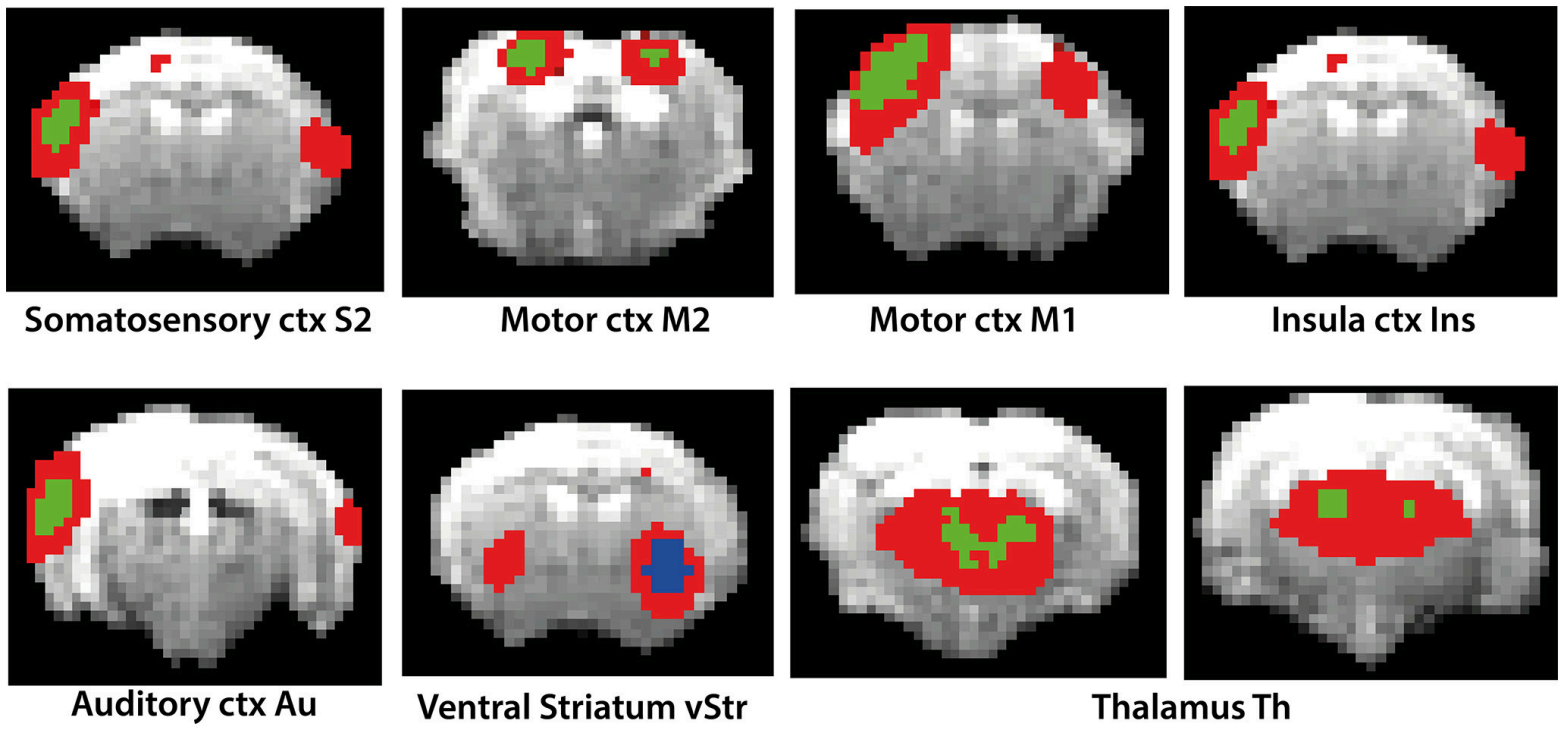
**Results of Dual Regression analysis for eight components derived from ICA (red color overlay)**. The reference of the components to anatomical structures is indicated in the figure. Bonferroni corrected DR results showing the regions whose co-activation with the ICA components (as shown in the figure) was significantly higher (green color) or lower (blue color) in isoflurane as compared to medetomidine-anesthetized mice.

DR revealed differences in functional connectivity between isoflurane- and medetomidine-anesthetized mice (Figure 2). Cortical areas display major difference between the two anesthetics, with mice under medetomidine anesthesia displaying only very weak intercortical functional connectivity.

The comparison of DR using probabilistic ICA based temporal concatenation with seed-based analysis using ICA informed seed selection revealed obvious similarities. Supplementary Figure 3 shows the comparison of seed-based with the ICA derived components. By definition the outcome of seed-based analysis is confined to network components associated with the selected seed region, typically revealing functional connectivity between homotopic regions. It is therefore not surprising that the model-free DR approach revealed additional anatomical regions as part of networks that displayed profound group differences when compared to the results of seed-based analysis. The previous study reports seed selection to include three sensory regions, the anterior (ant-), medial (med-), and posterior (post-) parietal cortex in addition to components in the cingulate cortex, ventral and dorsal striatum, and limbic areas (Grandjean et al., 2014). Data driven ICA also identified these regions as relevant components, but also additional regions including the olfactory tubercle, globus pallidus and amygdala, though among additional regions found only components involving the amygdala reached the significance level to be included in the results of the DR analysis.

### Between-network connectivity analysis using network modeling

Performing a between-network analysis on the basis of the DR results as described in FSLNets implies comparing consecutively the time series of network X as derived from ICA with the averaged time series of each of the other networks. In contrast, the seed-based approach compares a single time series signal from region X separately with the temporal signals from other regions. Hence, seed-based analysis is limited to the analysis of “within-network” connectivity, while DR-based FSLNets allows modeling of connectivity between network components in addition. This allowed grouping of individual components into functional networks (Table 1). ICAs were grouped together based on the FSLNets derived hierarchical clustering. We used a similar nomenclature as used by Liska et al. (2015) in order to keep the uniformity of reported networks. Five major networks have been identified: the default mode network (DMN) comprising cingulate cortex and hippocampus, the lateral cortical network (LCN) with somatosensory, secondary somatosensory, motor, and insular cortices, the associative cortical network (ACN) including auditory cortex, the subcortical network (SuCN) with piriform cortex, ventral striatum, and amygdala, and the thalamic network (ThN) comprising dorsal and ventral thalami.

**Figure 3:**
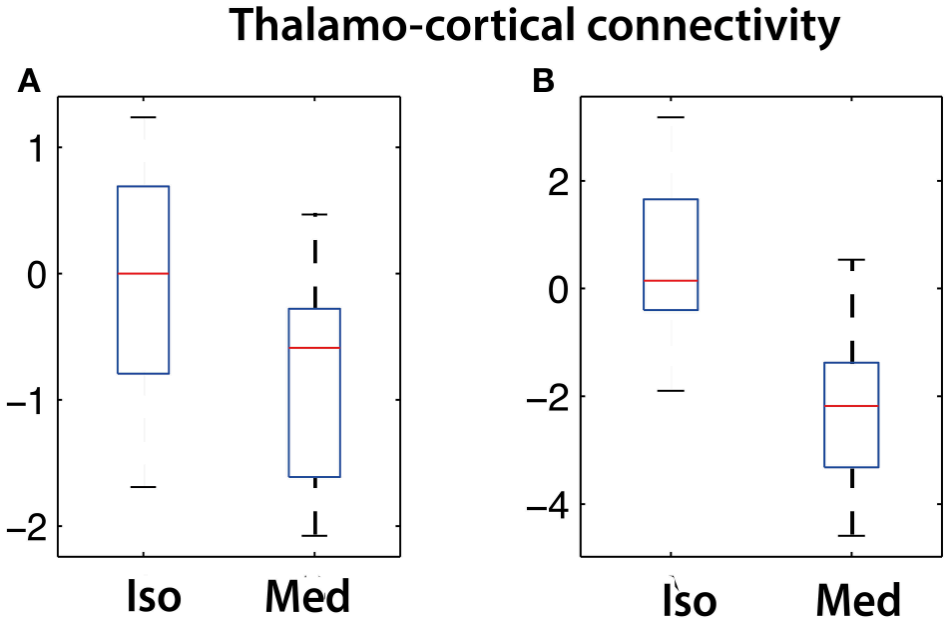
**Cortico-thalamic connectivity differences in isoflurane vs. medetomidine anesthetized mice**. Connectivity strength is shown for the network between **(A)** somatosensory cortex (S1) and ventral thalamus (vTh) and **(B)** between secondary somatosensory cortex (S2) and ventral thalamus (vTh). Under isoflurane anesthesia these connectivities are largely suppressed, while a significant negative correlation has been found for medetomidine-anesthetized mice.

**Figure 4:**
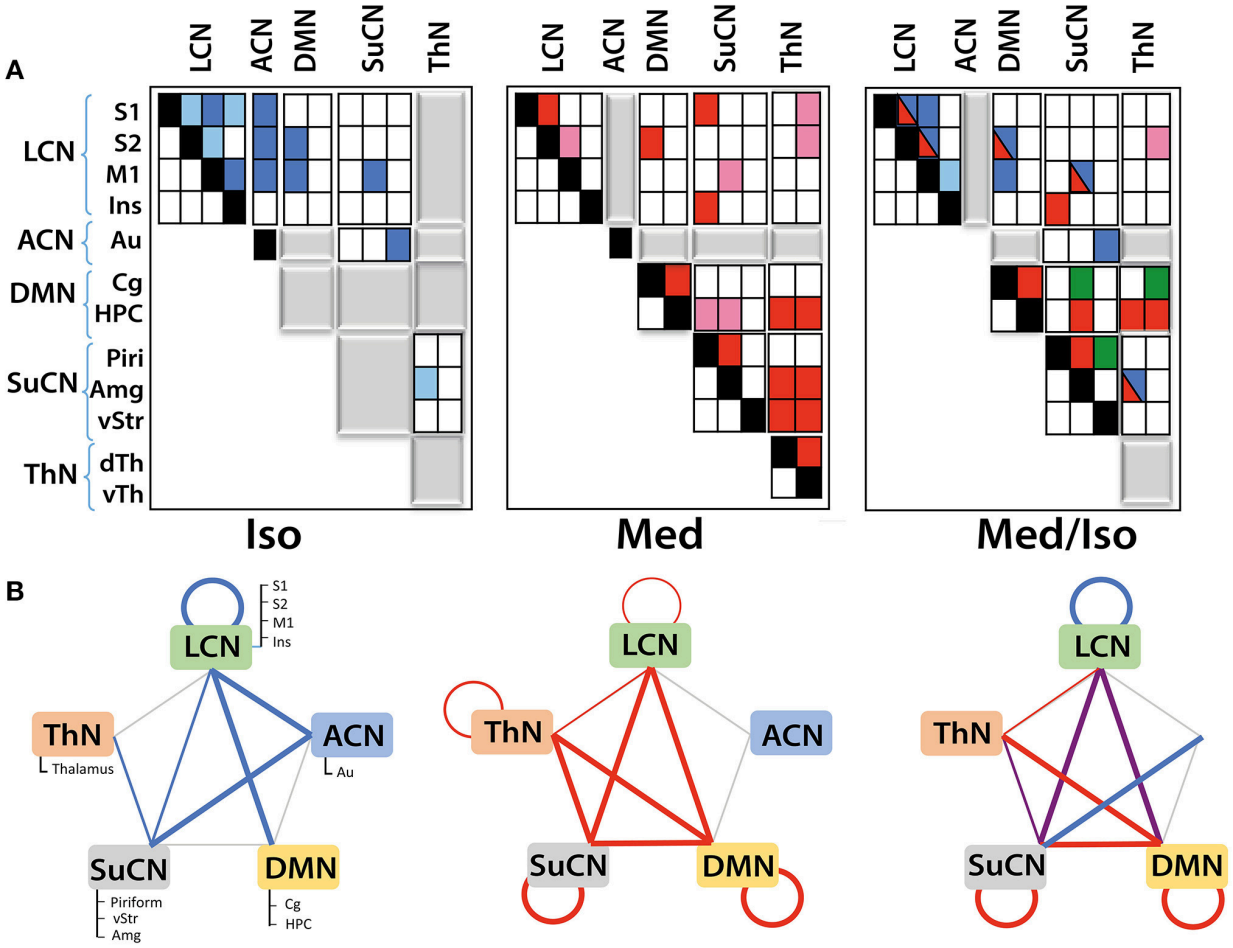
**Functional networks and their interaction as derived from DR analysis: (A)** Matrices displaying within- and between-network interactions under the various anesthesia regimens. Matrices have been structured according to the five functional networks identified. Colors indicate significant interactions observed under isoflurane (blue) or medetomidine anesthesia (red). For the group receiving the combination anesthesia, elements are indicated in different colors depending on whether they apparently arise from the isoflurane group (blue), from the medetomidine group (red), from both groups (blue/red) or were observed in the combination group exclusively (green). Positive correlations are indicated by dark colors, while light colors represent negative correlations. Gray blocks indicate the absence of any significant interaction. Significant group differences (with Bonferroni correction) in connectivity have been observed for all the interactions displayed. **(B)** Within- and between-network interactions detected in isoflurane (blue lines), medetomidine (red lines) and medetomidine/ isoflurane anesthetized mice. In isoflurane/medetomidine anesthetized mice network interactions, blue lines represent interactions observed in mice under isoflurane anesthesia only, red under medetomidine only, and violet represents interactions observed for both anesthesia regimes. The width of the lines in the figure indicates the strength of the connectivity.

The strengths of the connectivity between different network components was analyzed using network box plots as illustrated for the connectivity between S1-vTh and S2-vTh in isoflurane- vs. medetomidine-anesthesized mice (Figure 3). Thalamocortical interaction is completely suppressed in isoflurane-anesthetized animals, whereas under medetomidine anesthesia a weak negative thalamocortical correlation is observed. Analogous analyses have been carried out for all possible interactions among ICA components and for all anesthetic regimens, and the results displayed in the form of interaction matrices highlighting interactions found to be significant (Figure 4A; Supplementary Table 1 shows the connectivity values under different anesthetics between the selected ICA components obtained through boxplots).

Mice under isoflurane anesthesia displayed strong connectivity among LCN components, which was found to be less pronounced in medetomidine-anesthetized animals. In contrast the occurrence of interactions involving subcortical structures was a characteristic for medetomidine anesthesia. This included inter-thalamic connectivity as well as networks involving thalamo-cortical connections, both of which did not display significant functional connectivity under isoflurane anesthesia (Figure 4B). On the other hand, DMN-cortical connectivity was preserved under isoflurane anesthesia but not under medetomidine (Figure 4A, middle panel). Given the complementary nature of networks observed under these two anesthetics, Grandjean et al. (Grandjean et al., 2014) suggested the combination of the two as potentially attractive regimen.

The network interaction patterns observed when using the combination medetomidine/isoflurane can be largely represented as the superposition of the network interaction patterns obtained for each anesthetic alone (Figures 4A,B), with some deviations. All connections between cortical and subcortical structures observed under medetomidine were preserved for the combination regime except some interactions within the thalamic and the subcortical network. Also, the combination anesthesia displayed the strong intra-cortical networks observed for isoflurane but not for medetomidine. As Grandjean et al. (Grandjean et al., 2014) included propofol- and urethane-anesthetized mice in their study, the corresponding results of the network analysis for these two anesthetics have been compared to that obtained for isoflurane-anesthetized animals (Supplementary Figure 4).

The schemes described in Figure 4B capture the differential nature of network interaction only at a high level. When analyzing the interactions within and between modules in more detail, additional differences among the various anesthesia regimens become apparent (Figure 4A). For example, while both isoflurane-and medetomidine-anesthetized mice display within-LCN interactions the nature of these interactions is different: for the isoflurane group significant interactions between ICA components S1-S2, S1-M1, S1-Ins, S2-M1, M1-Ins were observed, while for medetomidine only two of these interactions (S1-S2, S2-M1) were found to be significant. For the combination regime medetomidine/isoflurane all interactions observed under isoflurane alone were found significant with the exception of S1-Ins. The connectivity within SuCN was found absent for isoflurane-anesthetized mice, but was found under medetomidine or medetomidine/isoflurane anesthesia. Inter-thalamic network interactions have been observed in medetomidine- but not in isoflurane- and medetomidine/isoflurane-anesthetized animals. Apart from inter-thalamic network connectivity, all the other interactions involving the thalamic network found under medetomidine were preserved in the combination regime. Similarly, the DMN was also found more functionally connected to other networks under the medetomidine and medetomidine/isoflurane combination regime than in isoflurane only. Some connectivity patterns have been observed exclusively under the medetomidine/isoflurane combination regime, such as the connection between ventral striatum and piriform cortex.

## Discussion

The vast majority of rodent fMRI studies involve the use of anesthesia, which inevitably interferes with brain function and may confound effects of interest unrelated to the anesthetic effects. Hence, understanding the effects imposed by the anesthetic regimen is essential for proper analysis of fMRI data, in particular information on functional connectivity across the brain. It may allow identifying anesthesia-specific network signatures, which might then be accounted for during further data analysis. The effect of anesthesia on functional connectivity in the rat and mouse brain has been investigated by several groups (Nasu et al., 2006; Masamoto et al., 2009; Jonckers et al., 2011, 2015; D'Souza et al., 2014; Schroeter et al., 2014; Pan et al., 2015; Grandjean et al., 2016). Grandjean et al. (2014) compared the effect of four different anesthetics on functional connectivity in the mouse brain using seed-based analysis and reported characteristic changes depending on the type of anesthetic used. In particular, functional connectivity patterns recorded under medetomidine differed from those observed for animals under isoflurane, propofol or urethane anesthesia. The current study using DR analysis confirmed these results for isoflurane and medetomidine and identified additional nodes/brain regions to be included in the anesthesia-specific signature. DR based network analysis including the analysis of between-network interactions is arguably a more comprehensive depiction of “systems-level” activity/connectivity in the brain, in particular with networks derived from a data-driven approach such as ICA, and thus might unveil new knowledge about brain systems where prior hypotheses are unclear. Functional connectivity between nodes could be either direct or indirect, i.e., relayed via another node. While correlation is a mere measure of functional connectivity, irrespective of its nature, PC analysis reveals direct connectivity exclusively (Smith et al., 2011). While Grandjean et al. (2014) have used FC analysis to identify regions displaying temporal signal profiles with high correlation to a seed region, in this work we employed PC measures to focus primarily on networks based on “direct” functional connectivity. An objective of network analysis is to identify nodes connected by direct connectivity (edges) and eliminate spurious edge effects due to an indirect third region in-between. It is important to note that direct connectivity does not imply monosynaptic connections. In fact, the structural correlate for direct FC can be both monosynaptic and polysynaptic. Our results reveal interesting within- and between-network interactions showing preserved intra- and inter-cortical interactions under isoflurane, subcortical interactions under medetomidine and superposition of these interactions under the combined anesthetics regimen.

DR and network analysis have mostly been applied to human studies so far with an exception of few recent reports in small animal rsfMRI (Henckens et al., 2015; Grandjean et al., 2016). On the other hand, the mouse brain and in particular its cortical organization is considerably simpler and less subject to inter-individual variability, which should add consistency to the data. In fact, DR yielded reasonable, neurobiologically plausible results for mouse rs-fMRI data. For example, this is illustrated by the fact that even for deep-lying small structures such as the amygdala or the ventral striatum (nucleus accumbens), statistically significant results have been obtained across groups.

The network interactions observed in mice receiving the medetomidine/isoflurane combination anesthesia can be largely composed as a superposition of networks found under the isoflurane or medetomidine alone, with some deviations. There are, nevertheless, a few aspects of the different group results that deserve special attention. DMN-ThN connectivity has been described as a structural connection in mice (Oh et al., 2014). In this study, we observed the functional links DMN-ThN and LCN-ThN in medetomidine, but not in isoflurane-anesthetized mice (Figure 3), which clearly highlights the potential confounds linked to the use of anesthesia in functional brain imaging studies. The anesthesia-specific connectivity pattern might arise from the different molecular modes of action of the two anesthetics or differential effects on the cerebrovasculature. Isoflurane is an anesthetic, while medetomidine is a sedative with analgetic activity. The two compounds have different molecular modes of action interacting with either the GABAergic (isoflurane) or the alpha2 adrenergic system (medetomidine). A striking observation is the loss of cortico-thalamic FC in isoflurane- and to a certain degree also in medetomidine-anesthetized mice. This may reflect the anesthetic efficacy of these drugs as loss of frontal-thalamic connectivity has been associated with loss of consciousness in humans (Akeju et al., 2014) and rats (Liang et al., 2015). The latter study demonstrated decreasing strength of this connection upon increasing the dose of the anesthetic. Along these lines, it has been demonstrated that light sedation with halothane (Sforazzini et al., 2014) or medetomidine (Nasrallah et al., 2014) preserved cortico-thalamic functional connectivity to some extent. Hence the observed differences in isoflurane- and medetomidine-anesthetized mice may reflect differences in anesthesia depth, i.e., the brain state. On the other hand, differences in the pharmacological mode of action and physiological activity (e.g., effects on the vascular tone) of isoflurane on medetomidine are likely to contribute to the differential responses. In addition, the two compounds exert rather opposing effects on the cerebrovascular system, isoflurane acting as vasodilator and medetomidine as vasoconstrictor, which may affect the translation of spontaneous neuronal activity into the BOLD signal assessed by fMRI. Interestingly, combining the two anesthetics at a low dose retained the interactions between DMN-ThN and LCN-ThN observed with medetomidine along with the intercortical interactions observed with isoflurane anesthesia, and thus constitutes an attractive anesthesia regimen for fMRI investigations in mice. Along similar lines, interactions within the SuCN between piriform cortex and amygdala were reliably detected in medetomidine- and medetomidine/isoflurane-anesthetized mice, but not under isoflurane only. This functional connectivity pattern is supported by the observation of structural connections between these regions (Oh et al., 2014). The existence of a structural connectivity does not warrant functional connectivity as illustrated by the differential functional connectivity patterns for the different anesthetics. For example, intracortical connectivity was found profoundly reduced in medetomidine- as compared to isoflurane-anesthetized mice. This connectivity was in part recovered when using the combination anesthesia, though the interaction remained weaker, in that a significant connectivity between LCN and ACN could not be detected anymore.

Mice under medetomidine anesthesia displayed anti-correlated functional connectivity between cortical structures and thalamus. There have been mixed reports on cortico-thalamic functional connectivity under anesthesia and it is widely debated in the literature. The preservation of thalamocortical activity under anesthesia has been reported previously in animals as well in humans (Mhuircheartaigh et al., 2010; Silva et al., 2010). Boveroux et al. (2010) reported similar anti-correlation during their study of propofol-induced unconsciousness in humans. An anti-correlated pattern of thalamus and cortical was also found in a study of rats with limbic seizures (Englot et al., 2009), in line with our results. On the other hand, some studies have shown no cortico-thalamic interaction during sedation (Alkire et al., 2000; White and Alkire, 2003; Zhao et al., 2008; Liu et al., 2013; Mashour and Alkire, 2013; Grandjean et al., 2014), while other studies have revealed diminished but detectable thalamo-cortical connectivity (Kim et al., 2012; Liang et al., 2012). It appears that the cortico-thalamic interaction is modulated by the type and depth of anesthesia, similar to response in other brain regions such as the frontal cortex displaying decreased activity in propofol and sevoflurane anesthesia in humans (Kaisti et al., 2002), a region differentially affected by anesthetics also in our study. Furthermore in (Smith et al., 2014), authors reported that DR outperforms seed-based analysis. Despite putting the seeds in the same areas identified by ICA, the authors were not able to replicate the results from DR, while their results from DR analysis had been independently verified in a separate group of subjects. This might explain the inability to detect statistically significant cortico-thalamic interaction in medetomidine-anesthetized mice in the previous study using seed-based analysis.

The results get even more complex when analyzing the interactions at the level of the individual ICAs that constitute a network. While for all anesthetic regimen tested, connections between the major networks have been observed—with the exception of ACN, for which interactions have been only detected under isoflurane anesthesia—there is considerable variability regarding the network components responsible for these interactions. These differences may again reflect anesthesia specific connectivity patterns. Alternatively, the differences found for the various anesthesia regimens may also arise from limitations in the statistical approaches, which—due to the small dimensions and correspondingly low SNR typically encountered in mouse fMRI—may lead to a significant finding for one but not for another anesthesia regime. An important limitation in fMRI studies assessing functional connectivity in anesthetized rodents is that data cannot be referred to the conscious baseline state. As a results, anesthesia induced changes in functional connectivity cannot be characterized. Nonetheless, FC pattern observed in anesthetized rodents have been found to correspond to patterns observed in awake humans (Pan et al., 2015). In addition, analysis of functional connectivity patterns under different anesthetics may help to identify anesthesia induced alterations in rs-fMRI patterns in mice.

Despite these limitations, the results obtained in this study are consistent with previous findings in humans and other species (Winters, 1976; Hudetz, 2012). The modulatory effects of anesthetics on functional connectivity between the brain regions highlights the importance of analyzing fMRI responses to pharmacological or physiological intervention at the level of brain networks rather than analyzing changes in isolated brain regions, a holistic approach that is gaining increasing attention in the neuroscience community.

In conclusion, we have used DR in combination with data-driven ICA analysis to study the effects of different anesthetic regimen on brain functional networks in the mouse. Five basic networks have been identified, which display within- and between-network interactions that depend on the anesthetic used. While medetomidine preserves most of the intra- and inter-network connectivities, except those involving the ACN, the intra- and inter-cortical network interactions (LCN-LCN, LCN-ACN) are better retained in isoflurane-anesthetized mice. An important result is that the network interactions observed under the combination anesthesia medetomidine/isoflurane largely constitute the superposition of the interactions found for each anesthetic alone. Understanding the differential effects of anesthetics on brain functional networks in animals is relevant when analyzing changes induced by physiological stress or pathological conditions. Deeper understanding of the effect of an anesthetic on large-scale brain networks is also relevant for clinical research, as it may help with achieving safer yet maximally effective anesthetic protocols with minimum side effects.

## Ethics statement

Data have been taken from a previous study that was carried out in compliance with the Swiss Law for animal protection. Protocols were approved by the Veterinary Office of Canton Zurich of Switzerland and this comes under the license number ZH 243/14.

## Author contributions

QB is the main author. He did the conception of the work, all the analysis, interpretation and writing. AS collected the data and contributed in the interpretation and writing. DC contributed in the writing. MR contributed in the interpretation and writing

### Conflict of interest statement

The authors declare that the research was conducted in the absence of any commercial or financial relationships that could be construed as a potential conflict of interest.
