# Supplementary material for: Resting State fMRI in Mice Reveals Anesthesia Specific Signatures of Brain Functional Networks and Their Interactions
Source: Front Neural Circuits. 2017 Feb 3;11:5. doi: 10.3389/fncir.2017.00005 (PMC5289996; doi:10.3389/fncir.2017.00005)
Supplement: Supplementary file 1 [file Table1.DOCX]

Supplementary Material

**Resting state fMRI in mice reveals anesthesia specific signatures of brain functional networks and their interactions**

Bukhari Q^1^, Schroeter A^1^, Cole D^1^ and Rudin M^1,2^

*** Correspondence:** Markus Rudin: rudin@biomed.ee.ethz.ch

# Supplementary Figures and Tables

## Supplementary Figure

**Supplementary Figure 1:** Examples of ICA components removed from further analysis as they mainly represent vascular structures, ventricles, tissue interfaces or could not be clearly assigned to anatomical structures (see labels in figure).

**Supplementary Figure 2**: Analysis Flowchart. The partial correlation matrix (1) is clustered in a subsequent step using hierarchical clustering algorithm (2) The input for hierarchical clustering were the time series in to hierarchical clustering, i.e. clustering is according to the functional similarity. Application of unpaired t-test yields enabled the identification of networks that displayed significant differences under the two anesthetic conditions A and B (3) as represented by network matrices containing corrected p-values for each contrast (A>B, A<B). For convenience, 1-p values are displayed. Elements below the diagonal are the 1-p values, elements above the diagonal indicate significance of the two-group t-test at corrected-p<0.05. The left matrix represents interaction with A>B and right matrix with B<A. The anatomical location of interacting networks components displaying significant differences is shown in (4), with red bars indicating positive and blue bars indicating negative correlation. The width of the bar indicates the strength of the correlation. The box plot (5) indicates the correlation coefficient for one specific network in condition A and B showing that the specific interaction was significant under A but not B.

**Supplementary Figure 3:** ICA derived components illustrating somatosensory cortex, ventral striatum, limbic areas, cingulate cortex and thalamus. For comparison with analogous seed-based regions the reader is referred to the Grandjean et. al. [1], Fig1.

**Supplementary Figure 4:** Comparison of functional networks found for urethane- and propofol-anesthetized mice compared to that of isoflurane-anesthetized animals. There is a striking similarity of the network maps for the three anesthetics. Nevertheless, there are few differences, e.g. some of the interactions appear weakened in urethane- and propofol- compared to isoflurane-anesthetized mice.

## Supplementary Table

**Suppl. Table 1**: Connectivity values between selected ICA components under isoflurane, medetomidine and medetomidine/isoflurane combined anesthetic regimens. The values were obtained from network box plots using unpaired t-test design matrix. Figures 4(a) and 4(b) displaying the network interaction matrix and graphs, respectively, is based on the quantitative information from these box plots.

|  | isoflurane | medetomidine | med/iso |
| --- | --- | --- | --- |
| S1 – S2 | -3.0 | 0.2 | 0.6 |
| S1 – M1 | 0.3 | 0 | 2.4 |
| S1 – Ins | -1.2 | 0 | 0 |
| S2 – M1 | -1.1 | -2 | 0.8 |
| M1 – Ins | 1.0 | 0 | -2 |
| Au – S1 | 0.5 | 0 | 0 |
| Au – S2 | 0.4 | 0 | 0 |
| Au – M1 | 0.8 | 0 | 0 |
| Cg – S2 | 1.5 | 1.3 | 1.1 |
| Cg – M1 | 1.2 | 0 | 0.7 |
| Amg – M1 | 1.4 | -1.3 | 0.5 |
| Piri – S1 | 0 | 0.4 | 0 |
| Piri – Ins | 0 | 1.8 | 0.1 |
| vTh – S1 | 0 | -0.7 | 0 |
| vTh – S2 | 0 | -2.1 | -0.4 |
| Au-vStr | 0.6 | 0 | 0.5 |
| Cg – HPC | 0 | 0.9 | 0.7 |
| HPC – Piri | 0 | -1.2 | 0 |
| HPC - Amg | 0 | -1.5 | 1.4 |
| HPC – dTh | 0 | 2.5 | 0.3 |
| HPC - vTh | 0 | 1.9 | 0.2 |
| Cg - Amg | 0 | 0 | 0.5 |
| Cg – vTh | 0 | 0 | 0.7 |
| Amg – dTh | -1.1 | 0.1 | 1.3 |
| Piri – Amg | 0 | 2.7 | 0.5 |
| Amg – vTh | 0 | 2.3 | 0 |
| vStr – dTh | 0 | 2.8 | 0 |
| vStr – vTh | 0 | 2.6 | 0 |
| Piri – vStr | 0 | 0 | 0.8 |
| dTh – vTh | 0 | 2 | 0 |
|  |  |  |  |
