# Supplementary figures and images for: Resting State fMRI in Mice Reveals Anesthesia Specific Signatures of Brain Functional Networks and Their Interactions
Source: Front Neural Circuits. 2017 Feb 3;11:5. doi: 10.3389/fncir.2017.00005 (PMC5289996; doi:10.3389/fncir.2017.00005)

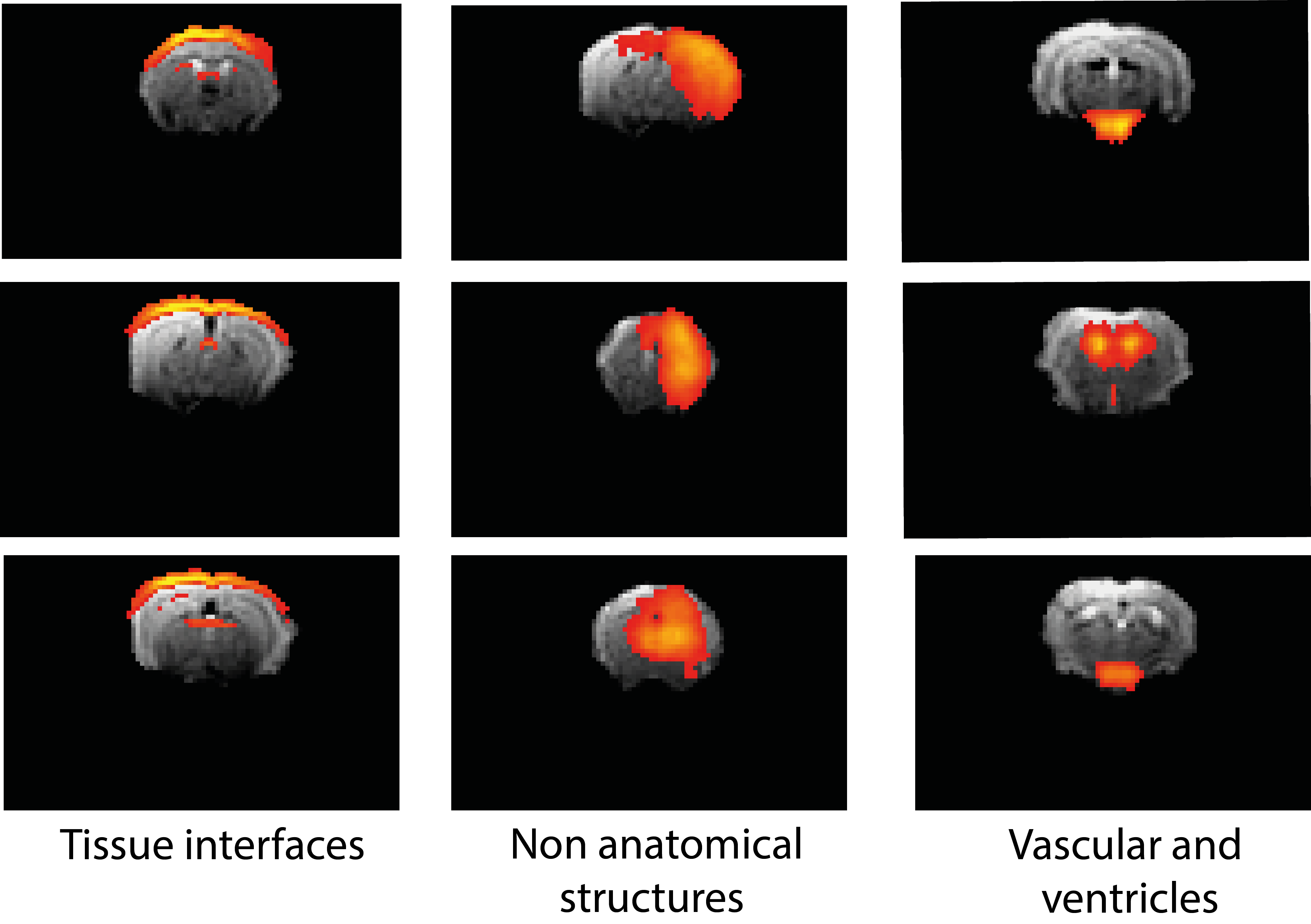

Supplement: Supplementary file 2 [file Image1.PNG]

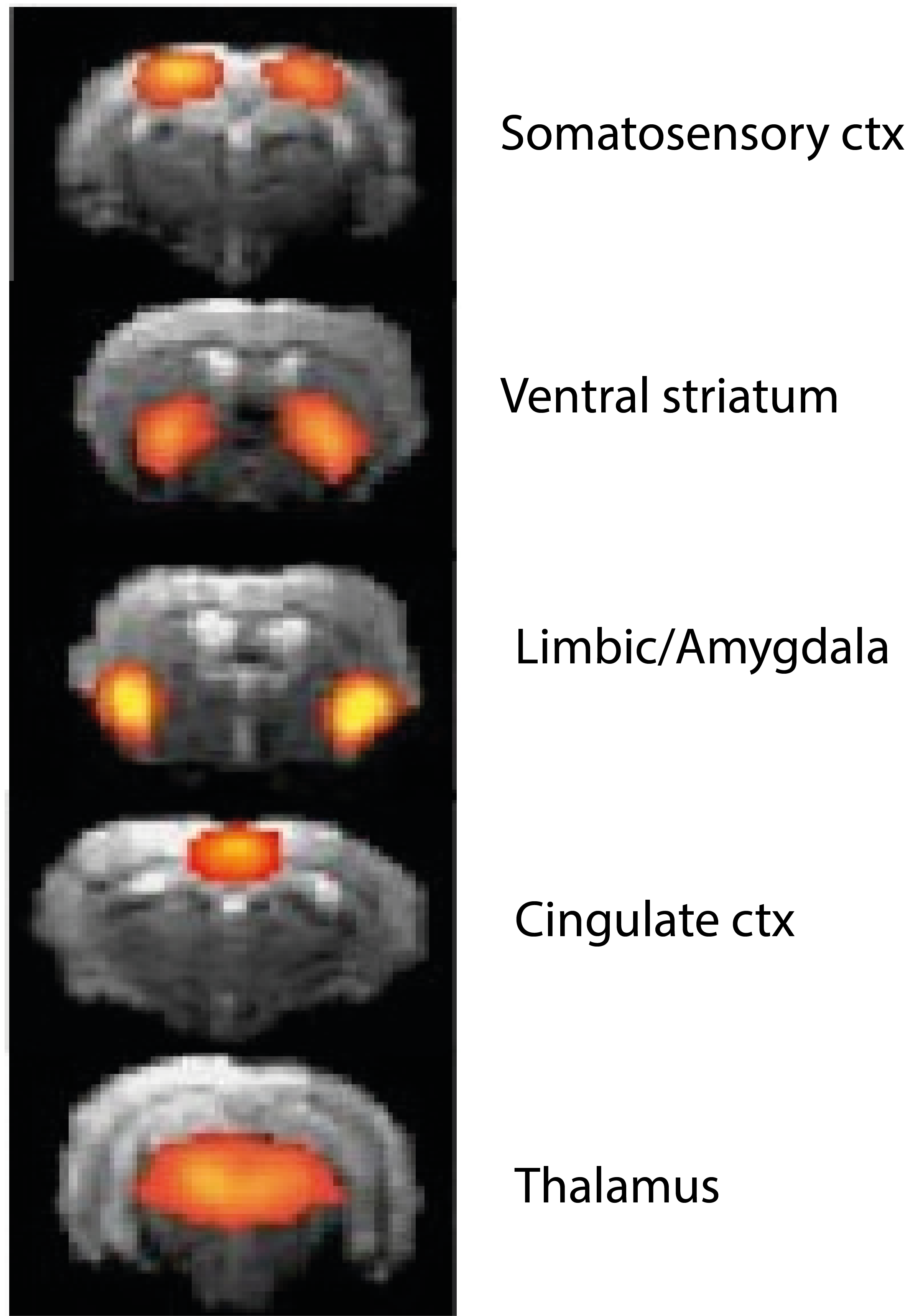

Supplement: Supplementary file 4 [file Image3.PNG]

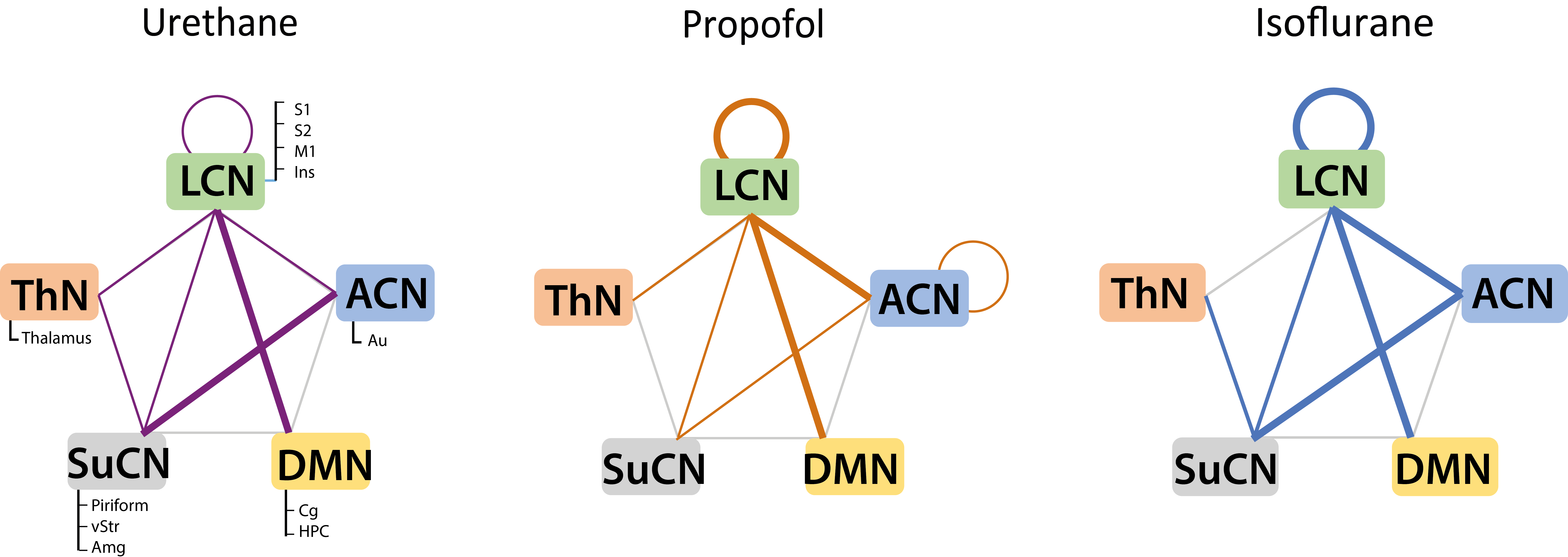

Supplement: Supplementary file 5 [file Image4.PNG]
